# Effects of posture on heart rate variability in non-frail and prefrail individuals: a cross-sectional study

**DOI:** 10.1186/s12877-023-04585-8

**Published:** 2023-12-19

**Authors:** Huiling Chen, Mimi Mun Yee Tse, Joanne Wai Yee Chung, Sui Yu Yau, Thomas Kwok Shing Wong

**Affiliations:** 1 School of Nursing and Health Studies, Hong Kong Metropolitan University, Sheung Shing Street, Ho Man Tin Hong Kong, China; 2 Kiang Wu Nursing College of Macau, Macau, China; 3 Hong Kong Nang Yan College of Higher Education, Hong Kong, China

**Keywords:** Posture, HRV, Frailty assessment, Difference

## Abstract

**Background:**

Frailty is an aging-related syndrome leading to high mortality in older adults. Without effective assessment and prevention of frailty, the incidence of frailty and relevant adverse outcomes will increase by 2050 as worldwide populations age. Although evidence suggested heart rate variability (HRV) is a potential measure of frailty, the role of HRV in frailty assessment remains unclear because of controversial findings. This study examined the effects of posture on HRV parameters in non-frail and prefrail individuals to understand the role of HRV in assessing frailty.

**Methods:**

Forty-six participants aged ≥ 50 years were recruited between April and August 2022. Frailty was defined using Fried’s criteria. HRV was measured in standing, sitting, and lying postures, respectively, using a Polar Watch, and analyzed using Kubios HRV Standard 3.5.0 (Kubios). The five most commonly used parameters were examined, including standard deviations of all normal-to-normal intervals (SDNN), root mean square of the successive differences (RMSSD), low frequency (LF), high frequency (HF), and LF/HF. Independent t-tests and Mann–Whitney tests were used for inter-group comparisons. Friedman tests were used for intra-group comparisons across postures.

**Results:**

The non-frail group showed significant differences in HRV parameters across postures (all *p* < 0.05), whereas the prefrail group did not demonstrate any difference (all *p* > 0.05). The differences in the non-frail group included higher RMSSD and HF in the lying posture compared to those in the standing posture (29.54 vs 21.99 *p* = 0.003, 210.34 vs 96.34 *p* = 0.001, respectively), and higher LF and LF/HF in the sitting posture compared to those in the lying posture (248.40 vs 136.29 *P* = 0.024, 1.26 vs 0.77 *p* = 0.011, respectively).

**Conclusions:**

The effects of posture on HRV were blunted in the prefrail group, which suggests an impaired cardiac autonomic functioning. Measuring the effects of posture on HRV parameters may contribute to frailty assessment. However, further evidence from larger cohorts and including additional HRV parameters is needed.

**Supplementary Information:**

The online version contains supplementary material available at 10.1186/s12877-023-04585-8.

## Introduction

Frailty is the most problematic expression of aging, leading to high mortality in older adults [1]. Around half of the individuals aged ≥ 60 years worldwide suffer from frailty (including being prefrail and frail) [2, 3]. This prevalence will increase by 2050 due to the rapidly aging populations (WHO, 2021). However, current instruments for frailty assessment are often subjective, and effective assessment is still needed [4, 5].

Heart rate variability (HRV) is a potential measure that may effectively assess frailty because of the interlinkage between frailty, the autonomic nervous system (ANS), and HRV [6, 7]. The impaired physiological functions and homeostasis in multiple systems in frailty syndrome are dominated by ANS function. The HRV, the beat-to-beat fluctuations in heartbeats, results from interactions between the sympathetic (SNS) and parasympathetic nervous systems (PNS), which are key components of the ANS including the cardiac autonomic nervous system (CANS). Given this interlinkage, the HRV could be an indicator for both ANS/CANS function and frailty assessment. Studies have reported differences in HRV between non-frail and frail individuals, suggesting that frail individuals had a lower HRV [8–10]. However, the results of these studies were controversial, leading to an unclear role of the HRV in assessing frailty.

Examining the effects of posture on HRV in non-frail and frail individuals may contribute to frailty assessment. HRV is a changing status which changes according to individual posture. Physiologically, HRV parameters change across standing, sitting, and lying postures because of changes in hemodynamics and their effects on ANS activity [11]. Although changing posture is a very common movement and would not lead to any adverse outcome for healthy young adults, it could be a stressor for prefrail and frail individuals because of orthostatic hypotension (OH) [12, 13]. OH is a symptom of ANS dysfunction in performing postural change, and it was associated with frailty and falls in older adults [12, 13]. In other words, because of OH and ANS dysfunction, the differences of HRV parameters across postures (i.e., standing, sitting, and lying) in the prefrail/frail individuals could be blurred or absent compared to that non-frail. Previous studies have examined the differences in HRV parameters across postures in healthy individuals [11, 14]; however, there is a lack of information on differences in non-frail and prefrail/frail individuals.

Consequently, the present study aimed to examine the effects of posture on HRV parameters in non-frail and prefrail individuals. The research questions were: Are there differences in HRV parameters across standing, sitting, and lying postures in non-frail and prefrail individuals? How do HRV parameters change across standing, sitting, and lying postures? This information may help to understand the role of HRV in assessing frailty and improve future frailty assessment. As a part of “The development and validation of a heart rate variability (HRV)-based model for frailty assessment of elderly community-dwelling Chinese” project, this study focused on the changing characteristics of HRV parameters across postures in non-frail and frail individuals.

## Methods

### Study design and participants

We performed a cross-sectional, preliminary study. Participants aged ≥ 50 years and able to walk (with or without a walking aid) were recruited from communities by convenience sampling between April and August 2022. Participants who had serious cognitive impairments, structural heart disease, or other serious diseases such as chronic obstructive pulmonary disease, cancer, or recent stroke were excluded. Those who had implanted cardiac pacemakers or were taking tricyclic antidepressants were also excluded. The minimum required sample size was estimated to be 30 according to the recommended sampling choice for a preliminary study [15, 16]. Considering the possible invalidity of the HRV recordings, a number greater than the minimum was recruited in this study. The study protocol was approved by the Research Ethics Committee (REC) of The Hong Kong Metropolitan University (REC No. HE-OT2021/04).

### Instruments

#### Physical frailty phenotype

The frailty level of the participants was measured using Fried’s criteria, including weight loss, exhaustion, physical activity, weakness, and slowness [17]. As a result of cultural differences across countries, there are over 70 modified versions of the Physical Frailty Phenotype (PFP) worldwide with different definitions of the five criteria and cut-off points [18]. This study adopted the modified PFP (mPFP) criteria by Wu et al., which were modified to meet the characteristics of Chinese populations based on the original PFP [19]. The mPFP was validated in the China Health and Retirement Longitudinal Study involving 28 provinces and 5301 older adults aged 60 years in China. Participants were classified as robust (non-frail), prefrail, or frail if they met none, one or two, or three or more of the criteria, respectively.

#### Polar watch

HRV data were collected using the Polar H10 chest belt and Polar Watch (Polar Ltd., Kwai Chung, Hong Kong). Polar H10 was designed as a heart-rate sensor and connects to a Polar Watch via Bluetooth. Vanderlei et al. reported that HRV data obtained from Polar products were as reliable as those from electrocardiograms (ECG) [20].

#### Procedures

Participants were instructed not to drink alcohol, coffee, or tea and not to exercise vigorously on the day before data collection. On the data collection day, the room temperature was confirmed to be comfortable. The study purpose and procedure were explained to the participants, and any questions from the participants were answered immediately. Written informed consent was obtained from those who were willing to participate in this study.

The participants were asked to complete the questionnaires by themselves, including demographics and the mPFP. The interviewer assisted the participants if they were unable to fill the questionnaire by themselves. After questionnaire collection, the participants were first taught to wear the Polar products, including the chest belt and watch. Second, the participants were instructed to stand, sit, and lie down for 5 min, respectively. The participants were told to breathe naturally during this period and not to talk except when necessary. HRV measurements were mostly taken during daytime (between 11:00 and 14:00). Seven participants were assessed at night (between 18:00 and 20:00) according to their availability.

### Data analysis

#### HRV data cleaning and selection

The HRV data were processed in Kubios HRV Standard 3.5.0 (Kubios, Kuopio, Finland). First, noise segments, defined as several consecutive abnormal beats, were manually excluded [21]. Second, individual abnormal beats were automatically corrected using the beat-correction function in Kubios. Then, an optimal 5-min HRV recording (without any noise) of each posture (i.e., standing, sitting, and lying posture) was selected for HRV analysis, as recommended by Hartikainen et al. [22]. Noisy recordings were excluded. Finally, parameter calculations were performed automatically by Kubios for each 5-min recording, including time-domain, frequency-domain, and nonlinear measures.

The five most commonly used parameters for studying HRV were chosen in this study [7]: standard deviations of all normal-to-normal intervals (SDNN, in ms), root mean square of successive differences (RMSSD, in ms), low frequency (LF), high frequency (HF), and LF/HF. These parameters have different clinical meanings. SDNN reflects the overall regulatory function of the ANS. RMSSD, and HF are used to estimate PNS activity. Both PNS and SNS may contribute to LF, but mostly the SNS does. LF may reflect baroreflex activity during resting conditions, and LF/HF is used to estimate the balance of activities between the SNS and PNS. Further details can be found in published literature, for example, in Shaffer’s and Pham’s reports [23, 24].

### Statistical analysis

All analyses were performed using SPSS 26.0 (IBM Corp., Armonk, NY, USA), and *p* < 0.05 was set as the significance threshold. First, a descriptive analysis was used for the demographic characteristics and risk factors. Second, baseline variances were compared between the groups. Fisher’s exact test and the Mann–Whitney U test were used to compare nominal and ordinal data, respectively. The scale data were compared using the independent t-test and Mann–Whitney U test according to the normality of data. Finally, the Friedman test was adopted to examine the differences in HRV parameters across postures in each group. If significant differences were found, pairwise comparisons were performed using a post-hoc analysis between the postures. The independent t-test and Mann–Whitney U test were applied to investigate the differences in HRV parameters of each posture between the two groups.

To examine the pure differences in the effects of posture on HRV in the non-frail and prefrail individuals, age, gender, comorbidities, and sample size distributions were controlled between two groups. Purposive sampling was used to select ten participants from the non-frail group whose age, comorbidities, and gender distributions were comparable with those in the prefrail group (Supplementary Table S1). Intra- or inter-group differences in HRV parameters across postures were further compared using the methods described above.

## Results

### Participants’ profile

Forty-six participants were recruited in the study, and 3 of them were excluded from data analysis due to excessive noises (over 80%) in their HRV recordings. According to PFP criteria, 33 participants were identified as non-frail, and 10 participants as prefrail. None of the participants were rated as frail. There were 37 females (86%) and 6 males (14%) in this study. The average age was 60.60 years (SD = 7.54). The average height and BMI were 158.71 (SD = 5.75) and 23.39 (SD = 2.94), respectively. The average systolic blood pressure (SBP) was 128.19 (SD = 18.55), and diastolic blood pressure (DBP) was 83.63 (SD = 12.03). Moreover, 14% of the participants had a smoking history, including past and present smokers; 23% drank alcohol occasionally. 19% of the participants had comorbidities and 51% had a medication history. Over half (58%) rated themselves as having good health; the others rated their health as normal or worse. All personal demographics and smoking and drinking history were compared between the groups. Apart from the age distribution, there was no difference between the two groups regarding personal demographics or relative histories. This indicated that the baseline characteristics were comparable between both groups. Further details can be found in Table 1.

**Table 1:** The baseline characteristics in non-frail and prefrail individuals, and comparisons between the two groups

	**Distribution of the sample**	**Characteristics within each group**		**Between-group difference**
		**Non-frail**	**Prefrail**	***p* value**
All sample^c^		33 (77%)	10 (23%)	
Gender^c^				0.127
Male	6 (14%)	3 (9%)	3 (30%)	
Female	37 (86%)	30 (91%)	7 (70%)	
Age (years)^a^	60.60 (7.54)	59.27 (6.55)	65 (9.23)	0.034
Height (cm)^a^	158.71 (5.75)	158.52(5.46)	159.35 (6.90)	0.692
BMI (Kg/m^2^)^a^	23.39 (2.94)	23.62 (2.78)	22.65 (3.45)	0.369
SBP (mmHg)^a^	128.19 (18.55)	127.79(20.62)	129.50 (9.58)	0.802
DBP (mmHg)^a^	83.63 (12.03)	83.06 (12.14)	85.50 (12.12)	0.581
Weight (Kg)^b^	58 (10)	58 (10)	57.35 (9)	0.505
Comorbidity^c^				0.070
YES	8 (19%)	4 (12%)	4 (40%)	
NO	35 (81%)	29 (88%)	6 (60%)	
Medication history^c^				0.281
YES	22 (51%)	15 (45%)	7 (70%)	
NO	21 (49%)	18 (55%)	3 (30%)	
Marital status^c^				0.206
Married	33 (77%)	27 (82%)	6 (60%)	
Unmarried/Divorced/Widow	10 (23%)	6 (18%)	4 (40%)	
Education^c^				0.419
Never been/primary school	15 (35%)	10 (30%)	5 (50%)	
Middle/technical/high school	24 (56%)	20 (61%)	4 (40%)	
College and above	4 (9%)	3 (9%)	1 (10%)	
Monthly income (HKD)^c^				0.810
> 10,000	37 (86%)	28 (85%)	9 (90%)	
≤ 10,000	6 (14%)	5 (15%)	1 (10%)	
Smoking history^c^				0.611
Yes	6 (14%)	4 (12%)	2 (80%)	
No	37 (86%)	29 (88%)	8 (20%)	
Drinking frequency^c^				> 0.999
Drinking occasionally	10 (23%)	8 (24%)	2 (20%)	
Never	33 (77%)	25 (76%)	8 (80%)	
Self-rated health^c^				0.273
Good	25(58%)	21 (64%)	4 (40%)	
Normal/worse	18 (42%)	12 (36%)	6 (60%)	

*SBP* Systolic blood pressure, *DBP* Diastolic pressure

^a^Described by [Mean (SD)]

^b^Described by [Median (IQR)]

^c^Described by [n (%)]

### Differences in HRV parameters across postures

Table 2 shows the differences in SDNN, RMSSD, LF, HF, and LF/HF across standing, sitting, and lying postures in the non-frail and prefrail groups. The five HRV parameters in the non-frail group were significantly different across standing, sitting, and lying postures, with χ^2^ = 8.42, *p* = 0.015 for SDNN; χ^2^ = 12.18, *p* = 0.002 for RMSSD; χ^2^ = 7.34, *p* = 0.025 for LF; χ^2^ = 13.78, *p* = 0.001 for HF; and χ^2^ = 16.78, *p* < 0.001 for LF/HF. Significant differences were also found for RMSSD and LF/HF in the non-frail group (*P* = 0.025 and *P* = 0.045, respectively) in the 10-case comparisons (Supplementary Table S2). In contrast, the prefrail group showed no significant difference in any of the parameters across postures (all *p* > 0.05).

**Table 2:** Differences of HRV parameters across postures in non-frail and prefrail individuals

	**Non-frail (*n* = 33)**						**Prefrail (*n* = 10)**			**Comparative difference in HRV of each posture between two groups (p value)**
	**Statistics**			**Pairwise comparison**^**a**^ **(*P* value)**			**Statistics**			
	**Median (IQR)**	**χ **^**2**^	***P* value**	**sd-ly**	**sd-st**	**ly-st**	**Median (IQR)**	**χ **^**2**^	***P* value**	
SDNN		8.42	0.015	0.147	0.014	> 0.999		3.80	0.150	-
Standing	24.02 (12.67)						21.82 (26.41)			0.832^c^
Sitting	28.37 (12.41)						34.13 (48.91)			0.166^b^
Lying	25.05 (11.93)						27.40 (33.27)			0.561^c^
RMSSD		12.18	0.002	0.003	0.029	> 0.999		0.60	0.741	-
Standing	21.99 (13.59)						27.25 (25.75)			0.226^c^
Sitting	26.90 (15.25)						38.42 (56.82)			0.237^c^
Lying	29.54 (16.87)						30.24 (37.53)			0.620^c^
LF		7.34	0.025	> 0.999	0.223	0.024		0.80	0.670	-
Standing	189.26 (401.47)						172.69 (342.98)			0.899^c^
Sitting	248.40 (291.63)						151.70 (921.76)			0.832^c^
Lying	136.29 (217.69)						150.30 (309.98)			0.921^c^
HF		13.78	0.001	0.001	0.127	0.290		1.40	0.497	-
Standing	96.34 (221.86)						188.75 (391.81)			0.125^c^
Sitting	195.66 (178.32)						330.21 (656.83)			0.286^c^
Lying	210.34 (247.38)						241.83 (746.89)			0.435^c^
LF/HF		16.78	< 0.001	< 0.001	0.886	0.011		1.40	0.497	-
Standing	2.02 (3.72)						1.21 (1.22)			0.093^c^
Sitting	1.26 (2.41)						0.57 (2.38)			0.141^c^
Lying	0.77 (1.15)						0.39 (1.30)			0.204^c^

*SD* Standing, *ST* Sitting, *LY* Lying, *SDNN* Standard deviation of all NN intervals, *RMSSD* Root mean square of the successive differences, *LF* Low frequency, *HF* High frequency

^a^Pairwise comparison across postures in the non-frail group;

^b^ results by independent t-test;

^c^ results by Mann–Whitney U test

HRV parameters for each posture were also compared between the non-frail and prefrail individuals. No significant differences were observed between the groups. However, compared to the non-frail, the prefrail individuals demonstrated higher HF and lower LF/HF in all three postures.

### The changing characteristics of HRV across postures

**Fig. 1:**
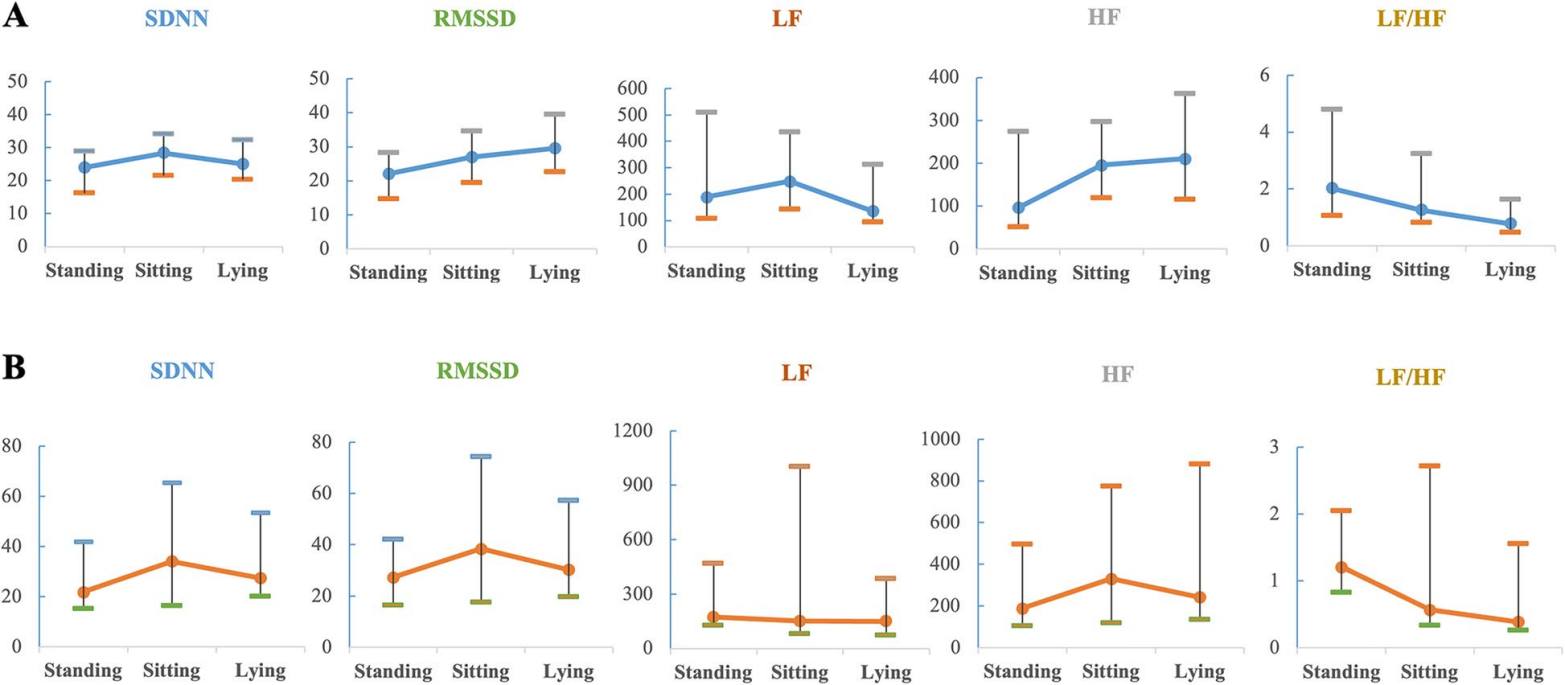
Variations of HRV parameters across postures in the non-frail (**A**) and prefrail (**B**) groups (Median [P_25_, P_75_])

Figure 1 depicts the changing characteristics of the HRV parameters across standing, sitting, and lying postures in the non-frail and prefrail groups. Generally, in the non-frail group, an increasing trend in PNS indices (RMSSD, HF) was observed from standing and sitting postures to the lying posture. The RMSSD and HF values in the lying posture were significantly higher than those in the standing posture, with *p* = 0.003 and *p* = 0.001 for RMSSD and HF, respectively. The median RMSSD in the sitting posture was significantly higher than that in the standing posture (26.90 vs 21.99, *p* = 0.029). A descending trend in median LF and LF/HF, which are more related to SNS activity, was demonstrated from the standing or sitting to the lying posture. The LF value was significantly higher in the sitting posture than that in the lying posture (248.40 vs 136.29, *p* = 0.024). The LF/HF values in the standing and sitting postures were significantly higher than that in the lying posture (*p* < 0.001 and *p* = 0.011, respectively).

Conversely, these change patterns were not observed in the prefrail group. For SDNN, representing both SNS and PNS activities, a similar trend (a decrease from sitting to lying down to standing) was found in the two groups. The difference between the standing and sitting postures was significant in the non-frail group (*p* = 0.014), while no significant difference was observed in the prefrail individuals.

## Discussion

Changes in SDNN, RMSSD, LF, HF, and LF/HF values across standing, sitting, and lying postures were examined in non-frail and prefrail individuals. The HRV parameters significantly differed across various body postures in the non-frail individuals. PNS-related parameters (RMSSD, HF) increased from standing to lying posture, and SNS-related parameters showed opposite trends. However, these changes were not observed in the prefrail individuals. Our results suggest poor adaptation of HRV parameters to postures in the prefrail individuals, suggesting impaired autonomic nervous function including CANS function.

Our results in the non-frail group were similar to those of previous reports on healthy individuals [11, 14, 25], and supported by Martinmaki et al. [26] who suggested stronger activity of the PNS and weaker activity of the SNS in the lying posture; reverse outcomes were observed in the standing posture. When sitting, the activities of the PNS and SNS were comparable. However, previous reports only focused on healthy individuals, and prefrail/frail or unhealthy individuals were not examined. Future studies should provide additional data on the effect of posture on HRV parameters in individuals with different levels of frailty.

Our findings in the non-frail individuals are supported by the body compensatory mechanism. Physiologically, the blood mass returning to the heart decreases in standing and sitting postures due to gravitational pull [11]. This reduction activates the body’s compensatory mechanism to prevent adverse events, such as dizziness or passing out, leading to an enhancement of sympathetic tone and a decrease in parasympathetic tone to increase cardiac output. Reverse activities occur when changing to a lying posture, seeking a balance in cardiac output due to the recovery process of the returned blood volume [11]. Hence, compared to the standing or sitting posture, higher HF and RMSSD and lower LF and LF/HF were observed in the lying posture due to the increase in parasympathetic tone, decrease in sympathetic tone, and decreased stimulation of peripheral baroreceptors [11].

The changing characteristics of HRV parameters across standing, sitting, and lying postures in non-frail individuals appear to almost agree with this body compensatory mechanism perfectly. Nevertheless, the lack of significant differences and specific changing characteristics in HRV parameters across postures in prefrail individuals suggests an impairment of cardiac autonomic functioning [7]. The differences in SDNN across postures is not discussed here because SDNN represents the activities of both SNS and PNS. Thus, it is impossible to distinguish whether the variations result from increased SNS activity or decreased PNS activity [26]. We can only reaffirm that the results regarding SDNN in the non-frail individuals in this study were similar to those demonstrated in healthy participants. In conclusion, the effects of posture on HRV parameters were blunted in the prefrail individuals, suggesting an impaired CANS function.

None of the parameters in each posture exhibited significant differences between the two groups (all P > 0.05). Similarly, Johnson reported no significant difference was found in sitting SDNN, RMSSD, LF, HF, and LF/HF among the non-frail, prefrail, and frail groups [27]. Katayama et al. did also not find significant differences in lying SDNN, RMSSD, and LF among non-frail, prefrail, and frail groups [9]. All these results suggest that there might be no significant differences in HRV parameters of each resting posture between non-frail and prefrail individuals. Higher HRV values could be observed in both non-frail and frail individuals because they do not always represent a state of health [23]. However, when evaluating changes of HRV across postures, CANS functioning in prefrail individuals was impaired.

The significant differences in the 10-case comparisons between the two groups further support this finding. In addition, HF and LF/HF might be promising parameters for frailty assessment, because they demonstrated similar results before and after baseline controlling. However, based on the small sample size, it is difficult to draw any definite conclusion.

### Limitations and future work

This study had some limitations that must be considered when interpretating the results. First, the data collection was performed during the COVID 19 pandemic, which lasted for three years. The access to public places including nursing homes and hospitals were inhibited by the Government. Within these constraints, the potential to recruit participants was severely limited, resulting in the small sample size and a lack of frail participants in this study. Nevertheless, we believe in the quality of our data and the reliability of our results because the data collection and processing were performed to the greatest extent possible following the recommendations of the available guidelines for HRV [21, 22, 28], and the 10-cases results further enhanced the reliability of our findings. However, evidence from larger samples including frail individuals is needed to verify our results. In addition, we used convenience sampling to recruit participants, which may have caused research bias. Even though we tried to reduce the selection bias and systematic error by strict entry criteria and double comparisons, random sampling is necessary for future studies.

Second, considering the time availability of the participants, a small number of participants were assessed at night and others during the day. Although this may have less impact on the changing characteristics of HRV parameters across postures, we still suggest that future studies collect HRV data at a fixed time, if conditions permit. Third, the resting HRV across postures was measured for 5 min, respectively, in this study. Although this duration is sufficient for HRV analysis, longer measurement durations are preferable for future studies to avoid potentially invalid HRV data. Fourth, although HRV data obtained by Polar Watch had a good agreement with those by ECG, Polar Watch may not detect all the signals due to loosened contact or poor connection. For future studies, we recommend researchers to collect HRV data using ECG if conditions permit [29]. Last, only five HRV parameters were examined in this study. Although they were the most commonly used ones, future studies should explore additional HRV parameters such as non-linear parameters.

## Conclusion

Our results suggest that the effects of posture on HRV in prefrail individuals are blunted compared to those in non-frail individuals. This indicates an impaired CANS function in prefrail participants. Measuring the effects of posture on HRV parameters could be effective strategy for frailty assessment. Further studies including large sample sizes, additional HRV parameters, and various frail participants (i.e., non-frail, prefrail, and frail) are still needed in this area.

### Supplementary Information

**Additional file 1: Supplementary Table S1. **The 10-cases baseline characteristics in non-frail and prefrail individuals. **Supplementary Table S2.** Differences of the HRV parameters across postures in non-frail and prefrail individuals (10-cases comparisons).

## Data Availability

The datasets used and/or analyzed during the current study are available from the corresponding author on reasonable request.

## Abbreviations

ANS: Autonomic Nervous System
CANS: Cardiac autonomic nervous system
ECG: Electrocardiogram
HRV: Heart Rate Variability
HF: High frequency
LF: Low frequency
mPFP: Modified Physical Frailty Phenotype
OH: Orthostatic hypotension
PNS: Parasympathetic Nervous System
PFP: Physical Frailty Phenotype
RMSSD: Root mean square of the successive differences
SDNN: Standard deviations of all normal-to-normal intervals
SNS: Sympathetic Nervous System
