# Correction: Effects of posture on heart rate variability in non-frail and prefrail individuals: a cross-sectional study

**DOI:** 10.1186/s12877-024-04851-3

**Published:** 2024-03-27

**Authors:** Huiling Chen, Mimi Mun Yee Tse, Joanne Wai Yee Chung, Sui Yu Yau, Thomas Kwok Shing Wong

**Affiliations:** 1 School of Nursing and Health Studies, Hong Kong Metropolitan University, Sheung Shing Street, Ho Man Tin, Hong Kong China; 2 Kiang Wu Nursing College of Macau, Macau, China; 3 Hong Kong Nang Yan College of Higher Education, Hong Kong, China

**BMC Geriatrics (2023) 23:870**

10.1186/s12877-023-04585-8

After publication of this article, the authors reported that in Table 1, under ‘Smoking history’ ‘Yes 6 (86%); No 37 (14%)’ should have read ‘Yes 6 (14%); No 37 (86%)”. Moreover, in Supplementary Table 1 ‘Never been/primary school 10 (35%); Middle/technical/high school 9 (56%); College and above 1 (9%)’ should have read ‘Never been/primary school 10 (50%); Middle/technical/high school 9 (45%); College and above 1 (5%)’.

The original article has been corrected.

