# Supplementary material for: Effects of posture on heart rate variability in non-frail and prefrail individuals: a cross-sectional study
Source: BMC Geriatr. 2023 Dec 19;23:870. doi: 10.1186/s12877-023-04585-8 (PMC10729458; doi:10.1186/s12877-023-04585-8)
Supplement: Supplementary file 1 — Additional file 1: Supplementary Table S1. The 10-cases baseline characteristics in non-frail and prefrail individuals. Supplementary Table S2. Differences of the HRV parameters across postures in non-frail and prefrail individuals (10-cases comparisons). [file 12877_2023_4585_MOESM1_ESM.pdf]

Supplementary Table S1 The 10-cases baseline characteristics in non-frail and prefrail individuals

	Distribution of the sample	Characteristics within each group		Between-group difference (p value)
		**Non-frail (n=10)**	**Prefrail (n=10)**	
Gender [c]				>0.999
Male	6 (30%)	3 (30%)	3 (30%)	
Female	14 (70%)	7 (70%)	7 (70%)	
Age (years) [a]	64.95 (7.67)	64.90 (1.98)	65 (9.23)	0.978
Height (cm) [a]	158.97 (7.39)	158.60 (2.59)	159.35 (6.90)	0.827
BMI (Kg/m2) [a]	23.03 (3.03)	23.41 (2.68)	22.65 (3.45)	0.587
SBP (mmHg) [a]	135.95 (16.59)	142.40 (19.91)	129.50 (9.58)	0.088
DBP (mmHg) [a]	86.80 (11.63)	88.10 (11.62)	85.50 (12.12)	0.630
Weight (Kg) [b]	55.85 (10.25)	55 (11.50)	57.35 (9.25)	0.853
Comorbidity [c]				>0.999
YES	7 (35%)	3 (30%)	4 (40%)	
NO	13 (65%)	7 (70%)	6 (60%)	
Medication history [c]				>0.999
YES	13 (65%)	6 (60%)	7 (70%)	
NO	7 (35%)	4 (40%)	3 (30%)	
Marital status [c]				0.087
Married	16 (77%)	10 (100%)	6 (60%)	
Unmarried/Divorced/Widow	4 (23%)	0 (0%)	4 (40%)	
Education [c]				0.853
Never been/primary school	10 (50%)	5 (50%)	5 (50%)	
Middle/technical/high school	9 (45%)	5 (50%)	4 (40%)	
College and above	1 (5%)	0 (0%)	1 (10%)	

Supplementary Table S1 (Continued)

	Distribution of the sample	Characteristics within each group		Between-group difference (p value)
		Non-frail (n=10)	Prefrail (n=10)	
Monthly income (HKD) [c]				0.739
>10000	17 (85%)	8 (80%)	9 (90%)	
≤ 10000	3 (15%)	2 (20%)	1 (10%)	
Smoking history [c]				>0.999
Yes	5 (25%)	3 (30%)	2 (80%)	
No	15 (75%)	7 (70%)	8 (20%)	
Drinking frequency [c]				>0.999
Drinking occasionally	5 (25%)	3 (30%)	2 (20%)	
Never	15 (75%)	7 (70%)	8 (80%)	
Self-rated health [c]				>0.999
Good	8 (40%)	4 (40%)	4 (40%)	
Normal/worse	12 (60%)	6 (60%)	6 (60%)	

Note: SBP = Systolic blood pressure; DBP = Diastolic pressure

[a] Described by [Mean (SD)]; [b] Described by [Median (IQR)]; [c] Described by [n (%)]

Supplementary Table S2 Differences of the HRV parameters across postures in non-frail and prefrail individuals (10-cases comparisons)

	Non-frail (n=10)						Prefrail (n=10)			Comparative difference in HRV of each posture between two groups (p value)
	Statistics			Pairwise comparison [a]			Statistics			
	Median (IQR)	χ^2	P value	sd-ly	sd-st	ly-st	Median (IQR)	χ^2	P value	
SDNN		4.20	0.122	-	-	-		3.80	0.150	-
Standing	17.64 (11.98)						21.82 (26.41)			0.436 [c]
Sitting	26.39 (21.23)						34.13 (48.91)			0.147 [b]
Lying	23.48 (9.91)						27.40 (33.27)			0.481 [c]
RMSSD		7.40	0.025	0.042	0.076	>0.999		0.60	0.741	-
Standing	19.12 (13.14)						27.25 (25.75)			0.089 [c]
Sitting	25.52 (23.60)						38.42 (56.82)			0.353 [c]
Lying	29.77 (9.87)						30.24 (37.53)			0.684 [c]
LF		2.92	0.232	-	-	-		0.80	0.670	-
Standing	128.59 (410.00)						172.69 (342.98)			0.353 [c]
Sitting	159.68 (359.12)						151.70 (921.76)			0.684 [c]
Lying	181.69 (288.98)						150.30 (309.98)			0.971 [c]
HF		4.15	0.145	-	-	-		1.40	0.497	-
Standing	67.24 (95.99)						188.75 (391.81)			0.019 [c]
Sitting	155.97 (107.87)						330.21 (656.83)			0.143 [c]
Lying	158.34 (125.47)						241.83 (746.89)			0.089 [c]
LF/HF		6.21	0.045	0.042	0.656	0.656		1.40	0.497	-
Standing	1.84 (3.14)						1.21 (1.22)			0.143 [c]
Sitting	1.73 (2.81)						0.57 (2.38)			0.218 [c]
Lying	1.26 (1.77)						0.39 (1.30)			0.089 [c]

Note: SD = standing; ST = sitting; LY = lying; SDNN = standard deviation of all NN intervals; RMSSD = root mean square of the successive differences; LF = low frequency; HF = high frequency

[a] Pairwise comparison across postures in the non-frail group; [b] results by independent t-test; [c] results by Mann-Whitney U test
